# Antimicrobial Potential and Chemical Characterization of Serbian Liverwort (*Porella arboris-vitae*): SEM and TEM Observations

**DOI:** 10.1155/2013/382927

**Published:** 2013-01-09

**Authors:** Amit Kumar Tyagi, Danka Bukvicki, Davide Gottardi, Milan Veljic, Maria Elisabetta Guerzoni, Anushree Malik, Petar D. Marin

**Affiliations:** ^1^Dipartimento di Scienze degli Alimenti, Università degli Studi di Bologna, Sede di Cesena, Piazza G. Goidanich 60, 47023 Cesena, Italy; ^2^Applied Microbiology laboratory, Centre for Rural Development and Technology, Indian Institute of Technology Delhi, New Delhi 110 016, India; ^3^Institute of Botany and Botanical Garden “Jevremovac”, Faculty of Biology, University of Belgrade, Studentski trg 16, 11000 Belgrade, Serbia

## Abstract

The chemical composition of *Porella arboris-vitae* extracts was determined by solid phase microextraction, gas chromatography-mass spectrometry (SPME GC-MS), and 66 constituents were identified. The dominant compounds in methanol extract of *P*. *arboris-vitae* were **β**-caryophyllene (14.7%), **α**-gurjunene (10.9%), **α**-selinene (10.8%), **β**-elemene (5.6%), **γ**-muurolene (4.6%), and allo-aromadendrene (4.3%) and in ethanol extract, **β**-caryophyllene (11.8%), **α**-selinene (9.6%), **α**-gurjunene (9.4%), isopentyl alcohol (8.8%), 2-hexanol (3.7%), **β**-elemene (3.7%), allo-aromadendrene (3.7%), and **γ**-muurolene (3.3%) were the major components. In ethyl acetate extract of *P*. *arboris-vitae*, undecane (11.3%), **β**-caryophyllene (8.4%), dodecane (6.4%), **α**-gurjunene (6%), 2-methyldecane (5.1%), hemimellitene (4.9%), and D-limonene (3.9%) were major components. The antimicrobial activity of different *P*. *arboris-vitae* extracts was evaluated against selected food spoilage microorganisms using microbroth dilution method. The Minimal Inhibitory Concentration (MIC) varied from 0.5 to 1.5 mg/mL and 1.25 to 2 mg/mL for yeast and bacterial strains, respectively. Significant morphological and ultrastructural alterations due to the effect of methanolic and ethanolic *P*. *arboris-vitae* extracts on *S*. Enteritidis have also been observed by scanning electron microscope and transmission electron microscope, respectively. The results provide the evidence of antimicrobial potential of *P*. *arboris-vitae* extracts and suggest its potential as natural antimicrobial agents for food preservation.

## 1. Introduction

Foodborne diseases mediated by food spoilage microorganisms are a major challenge in developing as well as developed countries [1]. Increasing concern about potentially harmful synthetic as compared with low mammalian toxicity and less environmental effects of the natural ingredients has led to their wide public acceptance [2]. In this regard, certain plant extracts or essential oils with notable antimicrobial activity can be used to delay or inhibit the growth of pathogenic and/or toxin producing microorganisms in food [3, 4]. Apart from higher plants, liverworts also contain certain natural compounds or derivatives potentially responsible for tubulin polymerization inhibitory, muscle relaxing, and antimicrobial activity. Liverworts are used as folk medicine worldwide [5].

Liverwort species are rich source of new secondary metabolites and produce a variety of sesquiterpenoids, possessing the bicyclo[4.3.0]nonane moiety such as acutifolone, bisacutifolone, pinguisenol, and chiloscyphone [6]. These natural products have unique structures including the cis-oriented continuous substitutions in the bicyclo[4.3.0]nonane structure. Although, several liverworts have been used as medicinal plants [7], only 10% of the liverworts have yet been studied systematically [5]. *Porella*, family Porellaceae (Hepaticae), consists of pungent and nonpungent leafy-stem liverworts widespread in the tropical, subtropical, and temperate regions of the world. Most *Porella* species are rich sources of sesqui- and diterpenoids [8, 9], many of which show interesting biological activities [10, 11]. To the best of our knowledge, there are no previous reports either on chemical characterization or on antimicrobial potential of *Porella arboris-vitae* liverwort.

In the present study, the chemical composition of *P. arboris-vitae* has been analyzed by SPME GC-MS. The antimicrobial activity of different extracts of *P. arboris-vitae *against selected food spoiling and pathogenic microorganisms has been evaluated. Further to investigate whether *P. arboris-vitae* extracts act by interfering the outer cell wall of bacterial cell, scanning electron microscopy (SEM) and transmission electron microscopy (TEM) have been used. Microscopic techniques provide the unique evidence of the antimicrobial potential of *P. arboris-vitae* extracts.

## 2. Material and Method

### 2.1. Chemicals and Plant Materials

Analytical grade chemical reagents (methanol, ethanol, and ethyl acetate used for livewort extraction were purchased from Sigma-Aldrich GmbH (Germany). For antimicrobial activity, the different dry extracts were dissolved in dimethyl sulphoxide (DMSO) (Sigma-Aldrich GmbH, Germany). For yeast and bacterial cultivation, yeast extract peptone dextrose (YPD) and tryptic soy broth (TSB) (Merck KgaA Darmstad, Germany) were used, respectively.

The samples of *Porella arboris-vitae *(With.) Grolle were collected from Derventa, Bajina Bašta (Serbia) in 2010. A voucher specimen (No. 16640) has been deposited in herbarium at Institute of Botany and Botanical Garden “Jevremovac,” University of Belgrade. Material was dried at room temperature for further use.

### 2.2. Extracts Preparation

Dried plants were pulverized into fine powder using an electric blender. Powdered material (5 g) was extracted with 100 mL of methanol, ethanol, or ethyl acetate for 24 h at room temperature. After 24 h, the mixture was filtered through Whatman filter paper No. 1. The solvents were evaporated from extracts using rotary vacuum evaporator (Laborota 4001, Heidolph) at 40°C. The yields of the extracts were 6.54%, 3.26%, and 1.28% for methanol, ethanol, and ethyl acetate extract, respectively. The obtained extracts were stored at 4°C for further observations.

### 2.3. Solid Phase Microextraction Gas Chromatographic-Mass Spectrometry (SPME GC-MS) Analysis

A divinyl benzene/carboxen/polydimethyl siloxane (DVB/CAR/PDMS) coated stable flex fiber (65 *μ*m) and a manual SPME holder (Supelco Inc., Bellefonte, PA, USA) were used in this study after preconditioning according to the manufacturer's instruction manual. Before each headspace sampling, the fiber was exposed to the GC inlet for 5 min for thermal desorption at 250°C.

Samples (5 mg) were put into sealed vials (20 mL) and then equilibrated for 10 min at 40°C. The SPME fiber was exposed to each sample for 10 min by manually penetrating the septum and the fiber was inserted into the injection port of the GC for 10 min for desorption. GC-MS analyses were carried out on an Agilent 6890 gas chromatograph (Agilent Technologies, Palo Alto, CA, USA) coupled to an Agilent 5970 mass selective detector operating in electron impact mode (ionization voltage, 70 eV). A Chrompack CP Wax 52 CB capillary column (50 m length, 0.32 mm i.d., 1.2 *μ*m df) was used (Chrompack, Middelburg, The Netherlands). The temperature program was 50°C for 0 min, then programmed at5°C/min to 230°C for 10 min. Injector, interface, and ion source temperatures were 250, 250, and 230°C, respectively. Injections were performed with a split ratio of 1 : 50 and helium (1 mL/min) as the carrier gas. Identification of chemical compounds was carried out by comparison of the mass spectra with mass spectra available on database of NIST05, WILEY8 libraries, and those of pure standards.

### 2.4. Microbial Strains

Different bacterial (*Salmonella* Enteritidis 155, *Escherichia coli* 555, and *Listeria monocytogenes* 56Ly) and yeast strains (*Saccharomyces cerevisiae* 635, *Zygosacharomyces bailii* 45, *Aerobasidium pullulans* L6F, *Pichia membranaefaciens* OC71, *Pichia membranaefaciens* OC70, *Pichia anomala* DBVPG3003, and *Yarrowia lipolytica* RO13) were obtained from the strain collection of the Dipartimento di Scienze degli Alimenti of Bologna University, Italy and used to evaluate the effect of liverwort extracts. Yeast strains were grown in YPD at 28°C for 48 h while bacterial strains were grown in TSB at 35°C for 24 h. After harvesting, microbial cells were suspended in sterile physiological water and used immediately.

### 2.5. Determination of Minimal Inhibitory Concentration (MIC) by Microdilution Method

In order to investigate the antimicrobial activity of the extracts, the modified microdilution technique was used [12]. MIC determination was performed by microbroth dilution technique using 96-well microtitre plates. The extracts were dissolved with 0.5% DMSO and added in TSB and YPD broth with bacterial (10^6^ cfu/mL) and yeasts (10^5^ cfu/mL) inoculum, respectively. The microplates were incubated either at 32°C for 48 h (bacteria) or at 28°C for 72 h (yeasts). The lowest concentrations without visible growth were defined as MIC. Further, Minimal Bactericidal Concentration (MBC)/Minimal Yeast-cidal Concentration (MYC) of the extracts have been evaluated. MBC/MYC values were defined as the minimal concentrations of extract not allowing the microbial growth on agar medium after a particular incubation at optimal temperature. DMSO was used as negative control.

### 2.6. Preparation of Samples for Scanning Electron Microscopy and Transmission Electron Microscopy

*S.* Enteritidis cells were incubated for 14 h in TSB at 30°C and 120 rpm. The suspension was divided into three portions. In two portions, methanolic and ethanolic *P. arboris-vitae* extracts at MIC level (1.25 and 1.25 mg/mL, resp.,) were added and third portion was left untreated as a control. These suspensions were incubated at 30°C for 8 h.

For investigating the effect of methanolic and ethanolic *P. arboris-vitae* extract, all the treated and untreated cells were harvested by centrifugation and were prefixed with a 2.5% glutaraldehyde solution overnight at 4°C. After this, the cells were again harvested by centrifugation and washed three times with 0.1 M sodium phosphate buffer solution (pH 7.2). Now each resuspension was serially dehydrated with 25, 50, 75, 90, and 100% ethanol, respectively. Then, cells were dried at “critical point.”

For SEM observation, a thin film of cells was smeared on a silver stub. The samples were gold-covered by cathodic spraying (Polaron gold). Morphology of the *S.* Enteritidis cells was observed on a scanning electronic microscope (ZEISS EVO 50). The SEM observation was done under the following analytical condition: EHT = 20.00 kV, WD = 9.5 mm, and Signal A = SE_1_.

For TEM observation, the pellet was postfixed in 1% osmium tetraoxide for 30 min, washed with phosphate buffer solution (pH 7.2), serially dehydrated in ethanol, and embedded in Epon-Araldite resin for making the blocks of the cells pellet. Ultrathin (50–100 nm) sections of *S.* Enteritidis cells were stained with uranyl acetate and lead citrate and observed under a Philips transmission electron microscope (CM-10) at 100 eV and direct magnification of 50.00 k.

### 2.7. Statistical Analyses

All the experiments were done in triplicate and repeatability was established. Significance of differences among treatments (*P* ≤ 0.05) was analysed using one way ANOVA (SPSS, 10.0 version).

## 3. Results and Discussion

### 3.1. Chemical Composition of Methanol, Ethanol, and Ethyl Acetate Extracts

More than 65 compounds of *P. arboris-vitae* were identified by the SPME GC-MS analysis of different extracts. The main components with their percentages and retention indices are listed in Table 1. In methanol extract of *P. arboris-vitae*, 43 components were identified, which represented 72.2% sesquiterpenes, 4.5% monoterpenes, 6.7% ketones, 0.5% nonterpene hydrocarbons, 1.87% aldehydes, 1.1% alcohols, and 11.7% others, about 98.6% of the total detected constituents. The major components were *β*-caryophyllene (14.7%), *α*-gurjunene (10.9%), *α*-selinene (10.8%), *β*-elemene (5.6%), *γ*-muurolene (4.6%), and allo-aromadendrene (4.3%). 38 components were identified from the ethanol extract of *P. arboris-vitae* which represented 59.5% sesquiterpenes, 4.8% monoterpenes, 2.5% nonterpene hydrocarbons, 19.6% alcohols, and 3.3% ketones, about 98.7% of the total composition where *β*-caryophyllene (11.8%), *α*-selinene (9.6%), *α*-gurjunene (9.4%), isopentyl alcohol (8.8%), 2-hexanol (3.7%), *β*-elemene (3.7%), allo-aromadendrene (3.7%), and *γ*-muurolene (3.3%) were the major components. 42 components were identified from the ethyl acetate extract of *P. arboris-vitae* which represented 46.8% nonterpene hydrocarbons, 37.6% sesquiterpenes, 6.8% monoterpenes, and 1.6% ketones where undecane (11.3%), *β*-caryophyllene (8.4%), dodecane (6.4%), *α*-gurjunene (6%), 2-methyldecane (5.1%), hemimellitene (4.9%), and D-limonene (3.9%) were the main compounds.

The presence of various monoterpenes,sesquiterpenes, andditerpenes in liverworts has been reported in earlier works [13–15]. These terpenes possess various kinds of biological activity and are beneficial for the health [16]. In the present study, *P. arboris-vitae* extract has shown the presence of many kinds of sesquiterpenoids which are ubiquitous in other liverworts. Higher percentage of sesquiterpenoids hydrocarbons could be responsible for higher antimicrobial activity.

### 3.2. Antimicrobial Activity

The MIC and MBC/MYC of different *P. arboris-vitae* extracts were determined against various bacterial (*S*. Enteritidis, *E. coli,* and *L. monocytogenes*) and yeast (*S. cerevisiae*, *Z. bailii*, *A. pullulans*, *P. membranaefaciens*, *P. anomala,* and *Y. lipolytica*) strains. These MIC and MBC/MYC values are shown in Table 2. Different *P. arboris-vitae* extracts exhibited concentration-dependent growth inhibition.

For the bacterial strains, the MIC varied from 1.25 to 2 mg/mL (Table 2). The MIC of different *P. arboris-vitae* extracts for Gram-positive bacteria (*L. monocytogenes*) was significantly (*P *≤ 0.05) lesser than Gram-negative bacteria (*S.* Enteritidis, *E. coli*), that is, 1.25 mg/mL and 2 mg/mL, respectively. However, MIC value of ethyl acetate extract for *L. monocytogenes *was significantly (*P* ≤ 0.05) higher than the methanol and ethanol extracts of *P. arboris-vitae*. The MBC of different extracts for bacterial strains varied from 2 to 3 mg/mL. MIC for streptomycin was 0.02–0.05 mg/mL and the MBC was 0.10 mg/mL. Higher MIC and MBC values of Gram-negative bacteria could be due to the presence of lipopolysaccharide cell envelope and highly hydrophilic cell membrane. Antimicrobial potential of 11 sesquiterpenoids, methanol and diethyl ether extract of Tahitian liverwort *Mastigophora diclados* was observed against *Staphylococcus aureus *NBRC 15035 and *Bacillus subtilis* NBRC 3134 where MIC of methanol and diethyl ether extract of *M. diclados* was 64 and 16 mg/mL, respectively [17]. These MIC values are quit higher than those evaluated in present study.

The MIC of methanol, ethanol, and ethyl acetate extracts for yeast strains varied from 0.5–1 mg/mL, 1–1.25 mg/mL, and 1–1.5 mg/mL, respectively (Table 2). The MIC values also varied as per the yeast strains. *Z. bailii, P. membranaefaciens *OC 71, *P. membranaefaciens* OC 70, and *P. anomala* DBVPG 3003 showed higher MIC than other tested yeast strains for ethanolic and ethyl acetate *P. arboris-vitae* extracts, while methanolic *P. arboris-vitae *extract showed lesser MIC for *P. anomala* CBS 5759 and *Y. lipolytica* than other tested strains. Similar pattern was found for the MYC of different extracts against yeast strains, that is, MYC of ethyl acetate extract ≥MYC of ethanolic extract >MYC of methanolic extract of *P. arboris-vitae*. The commercial antibiotic cycloheximide was used as a control, which possessed much lesser MICs (<0.05 mg/mL) than the different extracts against selected yeast strains. In general, different *P. arboris-vitae* extracts showed substantial antimicrobial activity and yeast strains were more sensitive than bacterial strains (Table 2).

Comparison of the MIC/MBC of different extracts for same bacterial or yeast strain showed that in general ethyl acetate extract had lower antimicrobial activity. This could be correlated with the presence of sesquiterpenes hydrocarbons which was higher in methanol (72.2%) and ethanol (59.5%) extracts than ethyl acetate extract (37.6%) of *P. arboris-vitae*. Earlier, Bukvicki et al. [18] also reported that high content of sesquiterpene components may account for the higher antimicrobial activity of methanol and ethanol extracts than ethyl acetate extract of liverwort. The presence of volatile sesquiterpene hydrocarbons such as *β*-selinene, *α*-guaiene, *α*-bisabolene, *α*-cedrene, caryophyllene, *α*-amorphene, *α*-chamigrene, bulnesene, and valencene acted synergistically to kill a broad range of plant and human pathogenic microorganisms [19]. In the present study, higher contents of these reported compounds were available in methanol and ethanol extract than the ethyl acetate extract of *P. arboris-vitae,* which support our results in regard to high antimicrobial activity of methanol extracts. Veljic et al. [20], also evaluated the antimicrobial activity of *Ptilidium pulcherrimum* methanol extract where the MIC for bacterial strains and fungal strains varied from 10–20 mg/mL and 0.5–2.5 mg/mL, respectively. These results are higher as observed in present study.

### 3.3. Morphological and Ultrastructural Alterations of **S*.* Enteritidis

#### 3.3.1. Scanning Electron Microscopy (SEM) Observation

**Figure 1:**
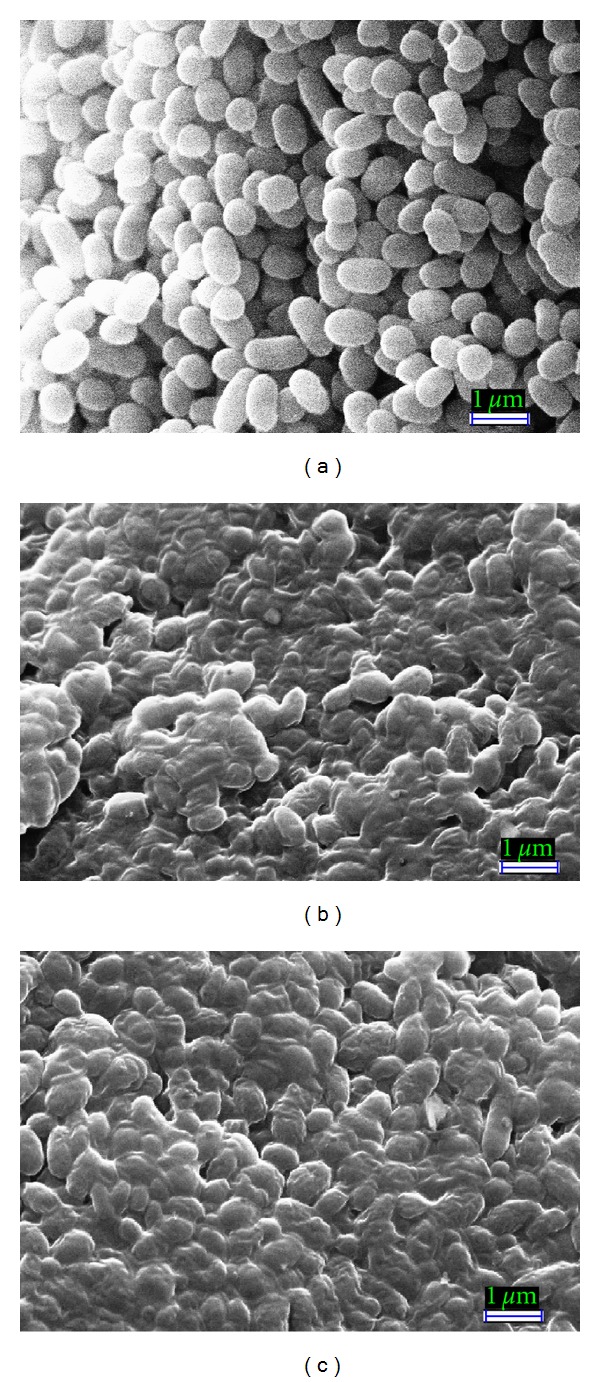
Scanning electron micrographs of untreated and treated *S.* Enteritidis cells. (a) Untreated cells with normal smooth surfaces (×20 K). (b) Shrinked aggregated and ruptured methanolic *P. arboris-vitae* extract treated cells (×20 K). (c) Shrinked aggregated and partially deformed ethanolic *P. arboris-vitae* extract treated cells (×20 K).

Bacterial cells treated with methanolic and ethanolic *P. arboris-vitae* extract at MIC level showed considerable morphological alterations in comparison to the control (Figure 1). Control *S.* Enteritidis cells appeared intact, separated from each other, turgid, and complete with smooth surface (Figure 1(a)) while methanolic and ethanolic *P. arboris-vitae* extract treated cells appeared to be aggregated and partially deformed (Figures 1(b) and 1(c)), respectively. SEM pictures revealed the complete loss of turgidity and the cytoplasmic material from the bacterial cells. It seems due to the leakage of cytoplasmic material of the bacterial cells and the aggregate cells appeared as sludge. Similar observations indicating the aggregations of bacterial cells as a stress response upon exposure to negative air ions have been reported earlier [21], where complete leakage of cytoplasmic material and loss of turgidity were found due to the combined exposure of negative air ions and lemon grass oil vapours. Other authors [22, 23] also observed aggregation of bacterial cells by the exposure to antimicrobial compounds similar to the present results. These SEM micrographs confirmed the evidences of antimicrobial potential of *P. arboris-vitae*.

#### 3.3.2. Transmission Electron Microscope (TEM) Observation

**Figure 2:**
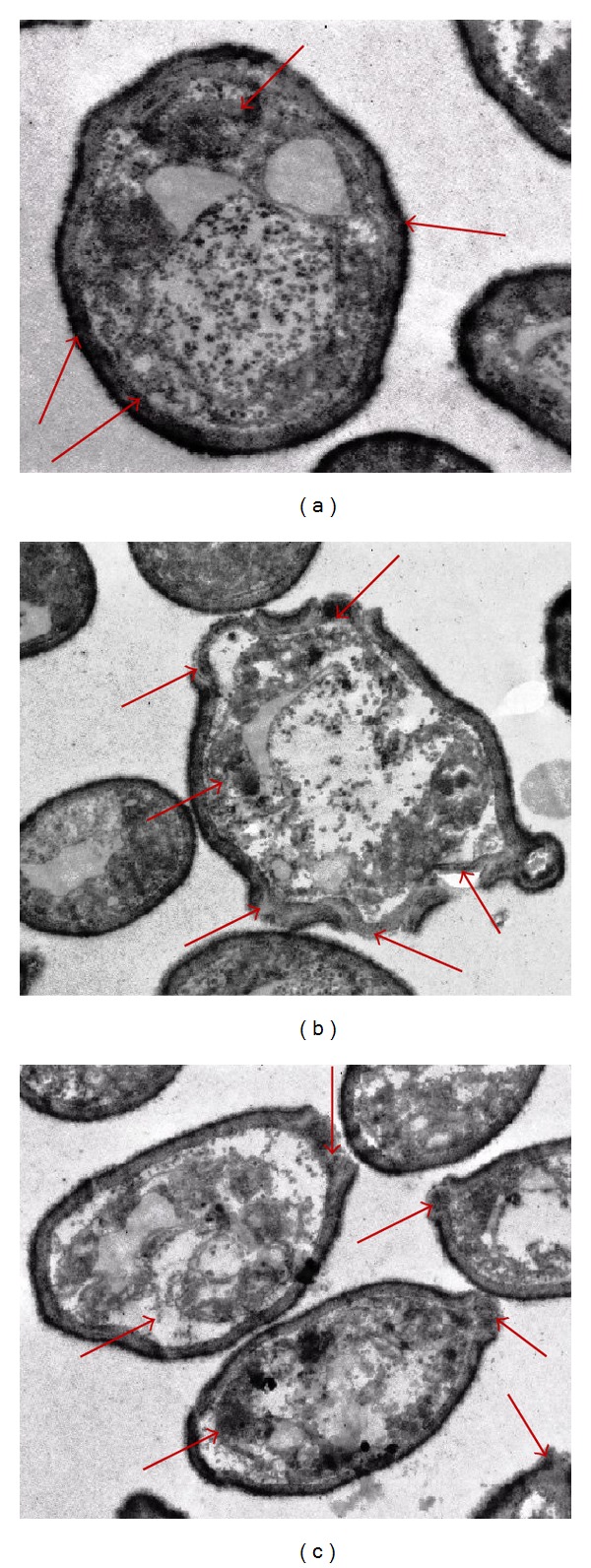
Transmission electron micrographs of untreated and treated *S.* Enteritidis cells. (a) Untreated cells having a regular outlined cell wall, plasma lemma lying closely to the cell wall, and regularly distributed cytoplasm (shown by arrows). (b) Methanolic *P. arboris-vitae* extract treated cells having extensive internal damage, unsymmetrical distributed cytoplasm, and larger and irregular periplasmic space (shown by arrows). (c) Ethanolic *P. arboris-vitae* extract treated cells having variable cell wall thickness appeared disrupted and variable periplasmic space (shown by arrows).

Further evidence of antibacterial potential of methanolic and ethanolic *P. arboris-vitae* extract has been obtained by TEM study (Figure 2). Untreated cells were studied as a control.

TEM photomicrographs of untreated *S.* Enteritidis cells show a regular outlined cell wall, plasma lemma lying closely to the cell wall (shown by arrows), and regularly distributed cytoplasm (Figure 2(a)). TEM photomicrographs of methanolic and ethanolic *P. arboris-vitae* extract treated bacterial cells revealed the variation in cell wall thickness and internal damages (Figures 2(b) and 2(c)). Methanolic extract treated bacterial cells (Figure 2(b)) showed extensive ultrastructural damages and wide range of abnormalities in comparison to ethanolic extract treated (Figure 2(c)) cells (shown by arrows). As shown in Figure 2, plasma lemma damaged and periplasmic space became larger and irregular in the treated cells. The cytoplasm appeared very dense at certain locations and unsymmetrically distributed in the cell (Figures 2(b) and 2(c)). At certain locations, the leakage of intracellular contents due to damage of cell envelop was also found (Figures 2(b) and 2(c)). This can also result from alteration in membrane permeability leading to draining out of the inner contents while the main structure of the outer membrane still remains intact.

Antimicrobial activity of various terpenes possesses discrete lipophilic characteristics and detectable water solubility may be potentiated by the fact that it can migrate across the aqueous extracellular medium, interact with, and damage lipid membranes. Since the outer layer of the Gram-negative bacteria is composed of lipopolysaccharide molecules and forms a hydrophilic permeability barrier providing protection against the effects of highly hydrophobic compounds [24], they may exhibit low sensibility of Gram-negative bacteria to the cytotoxic effect of the highly lipophilic monoterpenes. To the best of our knowledge, it is the first study where author tries to evaluate the ultrastructural changes in bacterial cells due to methanolic and ethanolic *P. arboris-vitae* extracts.

## 4. Conclusions

Antimicrobial potential of European folk medicinal plant *P. arboris-vitae* extracts against various bacterial and yeast strains has been investigated employing different microscopic techniques. SEM and TEM micrographs of the methanolic and ethanolic *P. arboris-vitae* extract treated bacterial cells together show the evidence of rupture, cell lysis, and loss of cytoplasmic material. SEM examination revealed that shrinkage of the bacterial cell was apparent in the cells treated with *P. arboris-vitae* extracts, when compared to the untreated ones. Loss of turgidity and leakage of the cytoplasm from the bacterial cells were also observed by TEM investigations.

Presence of different biologically active chemical constituents was characterized with SPME GC-MS and revealed the possibility to use the *P. arboris-vitae* extract with food materials. However, further investigations to determine the interaction of *P. arboris-vitae* extract with different food components are necessary. Antimicrobial activity of *P. arboris-vitae* extract should also be tested in real food systems for using it as a food preservative.

## Figures and Tables

**Table 1:** Chemical composition of *P. arboris-vitae* extracts.

Extracts		^ a^MeOH	^ b^EtOH	^ c^EtOAc
Monoterpene hydrocarbons				
D-limonene	1208	2.62	2.23	3.89
*β*-phellandrene	1220	1.07	1.67	0.00
p-cymene	1281	0.78	0.85	2.89

Total		4.47	4.75	6.78

Sesquiterpene hydrocarbons				
*α*-cubebene	1463	0.43	1.71	0.00
Cycloseychellene	1513	2.36	2.69	1.86
*β*-bourbonene	1519	1.45	1.25	0.00
*α*-gurjunene	1529	10.93	9.42	6.03
*β*-cubebene	1531	0.18	0.55	0.00
Cyperene	1534	0.52	0.00	0.00
D-longifolene	1550	0.54	0.96	1.42
*β*-elemene	1571	5.59	3.70	2.59
*β*-cubebene	1574	0.29	0.00	0.75
Neoisolongifolene	1588	2.75	1.85	0.80
*β*-caryophyllene	1594	14.70	11.75	8.34
*β*-gurjunene	1598	2.05	1.95	0.91
cis-thujopsene	1616	1.04	0.00	0.00
Amorpha-4,11-diene	1636	1.96	1.59	1.51
Allo-aromadendrene	1642	4.25	3.70	2.13
*β*-farnesene	1664	1.85	2.60	2.17
*γ*-muurolene	1671	4.59	3.27	2.47
Aonarene	1690	0.13	0.00	0.00
Aristolochene	1709	0.29	0.00	0.00
Germacrene D	1717	2.40	1.73	0.00
*α*-selinene	1724	10.76	9.57	4.21
*γ*-cadinene	1752	0.24	0.49	0.00
Selina-3,7(11)-diene	1763	0.26	0.00	0.93
*β*-bazzanene	1794	1.49	0.75	0.57
Calamenene	1855	1.15	0.00	0.87

Total		72.20	59.53	37.56

Nonterpene hydrocarbons				
2,6-Dimethylnonane	989	0.00	0.00	1.16
2,7,10-Trimethyldodecane	1005	0.00	0.00	0.36
5-Methyldecane	1035	0.00	0.00	1.85
2-Methyldecane	1038	0.00	0.00	5.10
3-Methyldecane	1048	0.00	0.00	4.56
Undecane	1091	0.23	0.86	11.27
1,2-Diethylcyclooctane	1128	0.00	0.00	0.28
2,3-Dimethyldecane	1142	0.00	0.00	0.75
Dodecane	1190	0.13	0.00	6.34
2-Phenylbutane	1260	0.00	0.00	0.15
Tridecane	1290	0.00	0.00	0.27
Hemimellitene	1292	0.00	0.00	4.91
p-Propyltoluene	1318	0.00	0.00	2.58
n-Butylbenzene	1322	0.00	0.00	0.49
2-Ethyl-p-xylene	1368	0.00	0.00	3.7446.82
3,5-Diethyltoluene	1402	0.00	0.00	0.53
Durene	1488	0.14	1.63	2.48

Total		0.50	2.49	

Alcohols				
1-Propanol	1031	0.00	1.53	0.00
1-Ethoxy-2-propanol	1168	0.00	3.02	0.00
Isopentyl alcohol	1203	0.00	8.75	0.00
2-Hexanol	1214	0.00	3.68	0.00
1-Isopropoxy-2-propanol	1244	0.00	2.35	0.00
1-Octen-3-ol	1441	0.88	0.24	0.00
1-(2-Methoxypropoxy)-2- propanol	1510	0.16	0.00	0.00
Trans-1,10-Dimethyl-trans-9- Decalinol	1859	0.07	0.00	0.00

Total		1.11	19.57	0.00

Ketones				
Methyl Isobutyl ketone	1007	1.00	0.84	0.00
3-Butyl-cyclohexanone	1124	0.00	0.00	1.06
4-Methyl-3-penten-2-one	1146	2.96	1.06	0.00
2,2,6-Trimethylcyclohexanone	1336	0.12	0.00	0.00
Cyclocolorenone〈epi-〉	2394	2.63	1.44	0.54

Total		6.71	3.34	1.60

Aldehydes				
Hexanal	1097	1.87	0.68	0.00
Furfural	1470	0.00	0.99	0.00

Total		1.87	1.67	0.00

Others				
Acetic acid	1448	7.75	7.09	3.33
Hexanoic acid	1840	1.97	1.69	0.37
Lanceol acetate〈Z-〉	1852	0.14	0.00	0.00
Caryophylene oxide	2019	1.84	0.95	0.84
Dodecanoic acid	2461	0.00	0.12	0.93
Octanoic acid	2083	0.00	0.00	0.13

Total		11.70	9.85	5.60

Total identified compounds		98.56	98.71	98.36

RI: retention index on CP WAX 52 CB capillary column; ^a^MeOH: methanol extract; ^b^EtOH: ethanol extract; ^c^EtOAc: ethyl acetate extract.

**Table 2:** Antimicrobial activity of liverwort *P. arboris-vitae* methanol, ethanol, and ethyl acetate extracts (mg/mL).

Microorganisms	*P. arboris-vitae* extract (mg/mL)						Control	
	MeOH		EtOH		EtOAc		Strep.	Cyclo.
	MIC	MBC/MYC	MIC	MBC/MYC	MIC	MBC/MYC	MIC	MIC
*S.* Enteritidis 555	1.5	2	1.5	2	2	3	0.05	—
*E. coli *155	1.5	2	1.5	2.5	2	3	0.05	—
*L. monocytogenes *56 Ly	1.25	2	1.25	2	1.5	3	0.02	—
*S. cerevisiae *635	1	1.5	1	2	1	2		<0.05
*Z. bailii *45	1	1.5	1.25	2	1.5	2.5		<0.05
*A. pullulans *L6F	1	1.5	1	2	1	2		<0.05
*P. membranaefaciens *OC 71	1	1.5	1.25	2.5	1.5	2.5		<0.05
*P. membranaefaciens *OC 70	1	1.5	1.25	2.5	1.5	2.5		<0.05
*P. anomala *CBS 5759	0.5	1	1	1.5	1.25	2		<0.05
*P. anomala *DBVPG 3003	1	1.5	1.25	2.5	1.25	2		<0.05
*Y. lipolytica *RO 13	0.5	1.5	1	1.5	1	1.5		0.02

MIC: minimal inhibitory concentration; MBC: minimal bactericidal concentration; MYC: Minimal Yeast-cidal Concentration (MYC); MeOH: methanolic extract; EtOH: ethanolic extract; EtOAc: ethyl acetate extract; Strep.: streptomycine; Cyclo.: cycloheximide.
